# Supplementary material for: Hybrid Models and Biological Model Reduction with PyDSTool
Source: PLoS Comput Biol. 2012 Aug 9;8(8):e1002628. doi: 10.1371/journal.pcbi.1002628 (PMC3415397; doi:10.1371/journal.pcbi.1002628)
Supplement: Text S4 — Complete source code for the PyDSTool package (version 0.88.120504). Includes API documentation and help files linking to web pages. This file is identical to the current public release on Sourceforge.net. (ZIP) [file pcbi.1002628.s004.zip › PyDSTool/html/PyDSTool.PyCont.BifPoint-module.html]

xml version="1.0" encoding="ascii"?


PyDSTool.PyCont.BifPoint


| Home | Trees | Indices | Help | PyDSTool |
| --- | --- | --- | --- | --- |

Package PyDSTool ::
Package PyCont ::
Module BifPoint

[hide private]

[frames] | no frames]

# Module BifPoint

source code

Bifurcation point classes. Each class locates and processes
bifurcation points.

Drew LaMar, March 2006


|  |  |
| --- | --- |
| Classes | [hide private] |

BifPoint

SPoint  
Special point that represents user-selected free parameter values.

BPoint  
Special point that represents boundary of computational domain.

BranchPoint  
Currently only works for EquilibriumCurve

FoldPoint

HopfPoint

BTPoint

ZHPoint

CPPoint

DHPoint

GHPoint

LPCPoint

PDPoint

NSPoint


|  |  |
| --- | --- |
| Variables | [hide private] |

\_classes = `['BifPoint', 'BPoint', 'BranchPoint', 'FoldPoint', ...`


|  |  |
| --- | --- |
| Variables Details | [hide private] |

### \_classes

Value:
:   ```
    ['BifPoint',
     'BPoint',
     'BranchPoint',
     'FoldPoint',
     'HopfPoint',
     'BTPoint',
     'ZHPoint',
     'CPPoint',
    ...
    ```

  


| Home | Trees | Indices | Help | PyDSTool |
| --- | --- | --- | --- | --- |

|  |  |
| --- | --- |
| Generated by Epydoc 3.0.1 on Fri May 4 15:24:03 2012 | http://epydoc.sourceforge.net |
